# The olfactory thalamus: unanswered questions about the role of the mediodorsal thalamic nucleus in olfaction

**DOI:** 10.3389/fncir.2015.00049

**Published:** 2015-09-18

**Authors:** Emmanuelle Courtiol, Donald A. Wilson

**Affiliations:** ^1^Emotional Brain Institute, Nathan Kline Institute for Psychiatric ResearchOrangeburg, NY, USA; ^2^Department of Child and Adolescent Psychiatry, New York University Langone Medical CenterNY, USA

**Keywords:** olfaction, mediodorsal thalamus, dorsomedial thalamus, piriform cortex, odor response

## Abstract

The mediodorsal thalamic nucleus (MDT) is a higher order thalamic nucleus and its role in cognition is increasingly well established. Interestingly, components of the MDT also have a somewhat unique sensory function as they link primary olfactory cortex to orbitofrontal associative cortex. In fact, anatomical evidence firmly demonstrates that the MDT receives direct input from primary olfactory areas including the piriform cortex and has dense reciprocal connections with the orbitofrontal cortex. The functions of this olfactory pathway have been poorly explored but lesion, imaging, and electrophysiological studies suggest that these connections may be involved in olfactory processing including odor perception, discrimination, learning, and attention. However, many important questions regarding the MDT and olfaction remain unanswered. Our goal here is not only to briefly review the existing literature but also to highlight some of the remaining questions that need to be answered to better define the role(s) of the MDT in olfactory processing.

## Introduction

The thalamus is a crucial crossroad structure in the brain that is recognized as a major contributor to the following functions: sensory perception, attention, sleep and arousal, memory, and cognition. Thalamic nuclei can be divided into (at least) two categories: first-order and higher order thalamic relays (Guillery, 1995). The first category, sensory recipient thalamic relays, processes information arriving from the periphery. The second category, higher order thalamic relays, processes information sent from many cortical areas. Higher order thalamic relays are key structures in cortico-thalamo-cortical networks (Sherman and Guillery, 2002; Mitchell et al., 2014; Saalmann, 2014).

The mediodorsal thalamic nucleus (MDT) is an example of a higher-order thalamic relay (Mitchell and Chakraborty, 2013). The MDT receives inputs from a wide variety of brain areas including cortical structures (notably the prefrontal cortex), brainstem structures, basal forebrain structures, and other thalamic nuclei (Groenewegen, 1988; Kuroda and Price, 1991a,b; Ray and Price, 1992; Guillery, 1995; Kuroda, 1998). In return, the MDT projects massively to the prefrontal cortex (Leonard, 1969; Krettek and Price, 1977a). The cytoarchitecture and the topographical distribution of the different inputs and outputs have led to the separation of the MDT into three subnuclei in the rat—medial, central, and lateral (Krettek and Price, 1977a; Groenewegen, 1988). The dense reciprocal connections between the MDT and the prefrontal cortex have placed the MDT as a critical structure in the study of cognitive processes.}In fact, lesions of the MDT in monkeys and rats are associated with a wide range of cognitive deficits: mnesic deficits, deficits in stimulus-outcome associations, deficits in representation of outcome value, and deficits in action-outcome association (Corbit et al., 2003; Mitchell and Gaffan, 2008; Ostlund and Balleine, 2008; Baxter, 2013; Mitchell and Chakraborty, 2013; Alcaraz et al., 2014; Mair et al., 2015). Electrophysiological recordings of the MDT have also demonstrated the contribution of the MDT in working memory, behavioral flexibility, goal-directed behavior, and stimulus reward-association (Oyoshi et al., 1996; Kawagoe et al., 2007; Yu et al., 2012; Han et al., 2013; Parnaudeau et al., 2013; Mair et al., 2015). The role of the MDT in cognition is thus increasingly well established (reviewed in Baxter, 2013; Funahashi, 2013; Mitchell and Chakraborty, 2013; Mitchell et al., 2014; Mitchell, 2015). In addition to these cognitive functions, the MDT also has a sensory component as the olfactory thalamus. As described below, there are firm anatomical and physiological data demonstrating the relationships among the olfactory cortex, the MDT, and the orbitofrontal associative cortex. These connections are particularly intriguing as they bring together one of the most phylogenetically oldest sensory systems with one of the more recently evolved cortical structures.

## Anatomy of the Olfactory Thalamus

**Figure 1:**
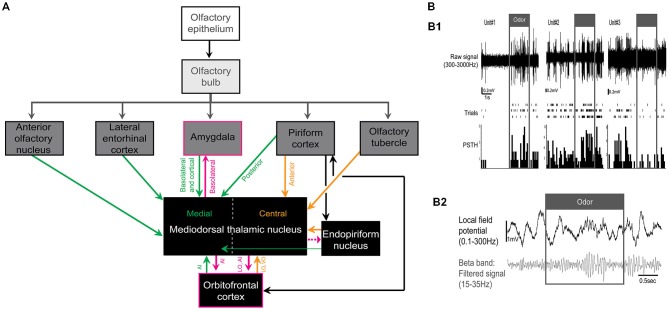
**(A)** Simplified schematic representation of the olfactory-related afferents and efferents of the mediodorsal thalamic nucleus (MDT) in rodents. Here we focused on the medial and central subnuclei of the MDT. Afferents in green and orange project mainly to the medial and central subnuclei of the MDT, respectively. Olfactory-related efferent projections of the MDT are outlined in pink (projection from MDT to endopiriform nucleus is represented with a dashed line because, to our knowledge, only one study has demonstrated this connection). LO and VO correspond to lateral and ventral orbital areas, respectively and AI to agranular insular areas. **(B)** Odor response in the MDT of urethane-anesthetized rats. **(B1)** Example of 3 odor responsive MDT units. From top to bottom: signal filtered between 300–3000Hz, raster plots of responses to the same odor presented three times and peristimulus time histograms (PSTH). Adapted from Courtiol and Wilson (2014). **(B2)** Example of odor-evoked local field potentials in the MDT. Top: local field potential filtered between 0.1–300 Hz and bottom: local field potential filtered in the beta band (15–35 Hz). Data used as an example here were described in Courtiol and Wilson (2014).

The thalamus is the major source of sensory information to the primary sensory cortex for all of the senses except olfaction. In fact, olfactory sensory neurons send their axons directly to the olfactory bulb which in turn projects to the primary olfactory cortex—a region including the piriform cortex, the anterior olfactory nucleus, the olfactory tubercle, the cortical nucleus of the amygdala, and the lateral entorhinal cortex (Price and Powell, 1971; Haberly and Price, 1977; Figure 1A). While there is no direct input from the olfactory sensory neurons to the thalamus, the MDT both receives and sends information to primary as well as secondary olfactory areas. An example of a major secondary olfactory area is the orbitofrontal cortex which has strong reciprocal connections with both the MDT and piriform cortex (Illig, 2005). While this review focuses on the MDT, the submedial nucleus of the thalamus also receives olfactory inputs (Price and Slotnick, 1983; Price, 1985).

### Olfactory Afferents

Powell et al. (1963) was one of the first to reveal the relationship between the olfactory pathway and the MDT by showing axonal fiber degeneration in the MDT following lesions in the piriform cortex. In addition to the piriform cortex, the MDT also receives direct input from the olfactory tubercle, the basolateral and cortical nuclei of the amygdala, the lateral entorhinal cortex, the anterior olfactory nucleus, the endopiriform nucleus, and the orbitofrontal cortex (Figure 1A). The MDT is thus the target of all the primary olfactory areas (e.g., piriform cortex) as well as some secondary olfactory areas (e.g., orbitofrontal cortex). Of note, the olfactory projections are topographical and involve two distinct subregions of the MDT: the medial and central subnuclei [in rats: (Powell et al., 1963; Heimer, 1968; Krettek and Price, 1974, 1977b; Inagaki et al., 1983; Price and Slotnick, 1983; Price, 1985; Cornwall and Phillipson, 1988; Kuroda and Price, 1991a,b; Kuroda et al., 1992a,b; Kowianski et al., 1999; Bay and Cavdar, 2013; Wilson et al., 2014); in hamsters: (Ferrer, 1969); Figure 1A]. The posterior piriform cortex, the anterior olfactory nucleus, the basolateral and cortical nuclei of the amygdala, the agranular insular areas, and the lateral entorhinal cortex project more medially in the MDT while the rostral piriform cortex (deep layers), the ventral and lateral orbital areas, the olfactory tubercle (polymorphic area), and the ventral endopiriform nucleus project mainly to the central region of the MDT [(Krettek and Price, 1974; Inagaki et al., 1983; Price and Slotnick, 1983; Price, 1985; Ray and Price, 1992; Bay and Cavdar, 2013); a small number of neurons from the endopiriform nucleus also project to the medial MDT (Cornwall and Phillipson, 1988)]. The detailed synaptic organization of the olfactory projections to the MDT is still poorly known. Kuroda et al. (1992a,b) described two types of axon terminals (large and small presynaptic terminals) from the piriform cortex to the central MDT which both appear to be excitatory. However, those two types of axon terminals still need to be characterized physiologically to identify whether or not the cells of origin differ. The targeting of specific cell types with cell-specific viral manipulations (e.g., optogenetics) may help to answer this question.

### Olfactory-Related Efferents

The MDT is known to be the origin of dense projections to the frontal cortex in the rat (Groenewegen, 1988). Furthermore, topographical projections from the MDT to olfactory-related structures, including the orbitofrontal cortex and the amygdala, have been established (Krettek and Price, 1977a; Ray and Price, 1992; Figure 1A). Interestingly, Kowianski et al. (1999) demonstrated that the endopiriform nucleus receives input from the MDT, though this has not been reported elsewhere.

In essence, the medial subnucleus of the MDT projects to prelimbic and dorsal agranular insular areas, as well as to the basolateral amygdala. The central subnucleus projects to the lateral part of the orbitofrontal cortex and the ventral part of the agranular insular area. Finally, the lateral subnucleus projects to the anterior part of the cingular area, the medial precentral area, and is reciprocally connected with orbital areas (Krettek and Price, 1977a; Groenewegen, 1988; Ray and Price, 1992). The projections of the MDT to the orbitofrontal cortex and basolateral amygdala are of great interest as those two areas are strongly involved in olfactory perception and odor-guided behavior [in rats: (Schoenbaum et al., 1999; Sevelinges et al., 2004; Feierstein et al., 2006; Roesch et al., 2007; Chapuis et al., 2009); in monkeys: (Tanabe et al., 1975; Rolls et al., 1996); in humans: (Jones-Gotman and Zatorre, 1993; Zald and Pardo, 1997; Gottfried and Zelano, 2011)].

The different anatomical studies provide strong evidence establishing the relationship between the olfactory pathway and the MDT. However questions related to the cells of origin and ultrastructural and synaptic organizations of the olfactory afferents in the MDT, as well as the neurotransmitters involved, still need to be investigated. Furthermore, given the recent demonstration that piriform cortical neurons projecting to orbitofrontal cortex may be non-randomly spatially organized, more detailed analysis of the olfactory cortex-MDT projection is warranted (Chen et al., 2014).

## Electrophysiological Studies of the Olfactory Thalamus

As a first step in understanding the contribution of the MDT in olfactory processing, it is important to characterize how olfactory information is encoded in the MDT. Here, we will describe the physiological responses of the MDT to olfactory stimulation. These data are based on responses recorded in the central and medial portions of the MDT.

First, evoked potentials and extracellular unitary responses in the MDT following the electrical stimulation of the olfactory bulb or lateral olfactory tract of various species have been described [in rats, central subnucleus: (Price and Slotnick, 1983; Price, 1985); in monkeys, medial subnucleus: (Benjamin and Jackson, 1974; Yarita et al., 1980; Takagi, 1986); in rabbits, medial subnucleus: (Jackson and Benjamin, 1974; Imamura et al., 1984)], with percentages of olfactory region-driven MDT units ranging from 16% to approximately 70% [(Imamura et al., 1984): 87/538 units in rabbits and Benjamin and Jackson (1974): approximately 180 units recorded in monkeys and 127 responsive units were localized in medial MDT].

Second, MDT units can respond to various odorant categories including biological, monomolecular, and mixture odorants [in rats, we observed odor-responsive units in medial, central and lateral MDT, but the boundary delimitation of the different subnuclei was not always clear and may account for the observation of odor-responsive units in the lateral MDT: (Courtiol and Wilson, 2014); in rabbits (Imamura et al., 1984); in monkeys: (Yarita et al., 1980; Takagi, 1986); in cats: (Motokizawa, 1974); Figure 1B1]. We observed that 51% of rat MDT units were odor-responsive, Motokizawa (1974) reported 44% of odor-responsive units in cats and Imamura et al. (1984) reported 55% of odor-responsive units in rabbits, although this last percentage was calculated only on MDT neurons responding to lateral olfactory tract shocks (48/87). The tuning of MDT neurons to odorants seems to be dependent on the species, odorant set, and level of anesthesia used. In fact, Courtiol and Wilson (2014) reported that, in anesthetized rats, 63% of units responded to only one odor out of the odor set used. Yet, this tuning has been shown to be more broad in awake monkey and anesthetized rabbits, with 41.5% of MDT neurons responding to four odors and more than 80% responding to two odors or more, respectively (Yarita et al., 1980; Imamura et al., 1984). Nevertheless, two common features of these reports are that MDT units: (1) primarily display excitatory activities in response to odors although suppressive responses can also be observed (Figure 1B1) and (2) are similarly responsive to biological, monomolecular or mixture odorants. In addition, our work and the work of others reported that some MDT units can display respiration-linked activity (Imamura et al., 1984; Courtiol and Wilson, 2014). Interestingly, at least in anesthetized rabbits and unanesthetized monkeys, the majority of odor responsive neurons are sensory specific as they do not respond to visual, auditory nor somatic stimulation (Yarita et al., 1980; Imamura et al., 1984).

Finally, the response of the MDT to odorant stimulation can also be recorded at the network level. In recording the local field potentials in the MDT of urethane-anesthetized rats (Courtiol and Wilson, 2014; Figure 1B2), we observed that odorants induce the conjoint emergence of beta frequency oscillations in the MDT and piriform cortex. Interestingly, a subset of MDT units fire in phase with the beta frequency oscillations recorded in the piriform cortex. These beta oscillations may offer an effective mechanism for olfactory information transmission between the piriform cortex and the MDT (Tallon-Baudry et al., 2001).

Taken together, these studies reveal that the MDT can respond to and encode odorant information in a manner similar to other primary and secondary olfactory structures. However, future studies will need to determine: (1) the contribution of each of the olfactory inputs to the MDT response; (2) the impact of the MDT on downstream targets such as the orbitofrontal cortex; and (3) given the rich variety of non-olfactory inputs to MDT, how MDT neurons contribute to multi-sensory associations and contextual effects on odor perception. Performing multi-site unitary recordings and using a large set of odorants in behaving animals may achieve this.

## The Still Unclear Role of the MDT in Olfaction

Beyond its basic odor responsiveness, the role of the MDT in olfaction remains unclear. In this last section, we will review the different studies involving the MDT in olfaction, point to some common threads among the available literature, and highlight remaining questions.

Studies of the effect of damage to the MDT in both humans and animal models have provided some useful information about its role in olfaction (Tham et al., 2009). The results of these studies have demonstrated that both humans and animal models with MDT damage are not anosmic and do not present deficits in olfactory detection [in rats and hamsters: (Eichenbaum et al., 1980; Sapolsky and Eichenbaum, 1980); in humans: (Potter and Butters, 1980; Sela et al., 2009)]. Furthermore, humans with Korsakoff’s disease presenting MDT damage are not anosmic but odor detection effects vary between studies, probably depending on the extent of the damage (Jones et al., 1975; Potter and Butters, 1980; Pol et al., 2002). While the results of those studies have shown that MDT damage does not affect olfactory detection, they support the fact that MDT lesions do affect other olfactory functions including olfactory perception, discrimination, learning, and attention. In respect to olfactory perception, Sapolsky and Eichenbaum (1980) demonstrated that MDT-lesioned hamsters show distorted odor preference, i.e., less interest in female and male odors and reduced preference for genital sniffing, leading to maladaptive sexual behaviors. Altered odor preference was also reported in humans. In fact, patients with damage in the MDT present altered olfactory hedonic perception (Rousseaux et al., 1996; Asai et al., 2008; Sela et al., 2009). These results are interesting given the reciprocal connections between the MDT and the amygdala, as the neuronal activity in the amygdala has been shown to be directly influenced by the hedonic valence of olfactory stimuli in humans (Zald and Pardo, 1997). The MDT may thus be part of a network, including the amygdala, involved in the coding of the hedonic valence of the olfactory stimuli.

With respect to discrimination, Eichenbaum et al. (1980) showed that rats with lesions of the MDT exhibit deficits in difficult odor discriminations. For instance, rats with MDT lesions need more trials to reach the discrimination criterion when the task difficulty is increased by using either novel stimuli or perceptually similar stimuli (Eichenbaum et al., 1980; Slotnick and Risser, 1990). However, these deficits can be temporary and alleviated if the animals receive intensive training (Staubli et al., 1987). Deficits in odor discrimination, as well as in odor identification, were also reported in humans with thalamic damage (Sela et al., 2009; Tham et al., 2011a). Notably, Tham et al. (2011b) used both olfactory and visual discrimination tests and demonstrated that patients with MDT damage presented impaired performance selectively for olfactory discrimination compared to visual discrimination. The effects of MDT lesions extend beyond olfactory discrimination given that deficits in olfactory learning have also been observed. For example, rats with central MDT lesions perform as well as controls for preoperatively learned visual discrimination tasks and in the acquisition of a simple go/no-go odor discrimination task (Slotnick and Kaneko, 1981). However MDT-lesioned rats were impaired when performing odor reversal learning. The authors also noticed in a set of preliminary experiments that three MDT-lesioned rats were not impaired in their acquisition of a visual discrimination reversal set suggesting that the deficits were modality specific. Thus, lesions of the MDT seem to induce a severe deficit in reversal learning and the degree of impairment seems to be related to the extent of the lesion (Slotnick and Kaneko, 1981; Staubli et al., 1987; Lu and Slotnick, 1990; Slotnick and Risser, 1990; McBride and Slotnick, 1997). Importantly, one of the reciprocally connected structures of the MDT—the orbitofrontal cortex—has also been involved in reversal learning (Roesch et al., 2007). The MDT-orbitofrontal cortex network may integrate stimulus-outcome associations to flexibly guide goal-directed behavior.

Rats with thalamic lesions which include the MDT also present deficits in an olfactory continuous delayed nonmatching-to-sample task with no effect on an odor discrimination task. Although, when lesions were more restricted to the MDT, the deficits were minimal in this task (Koger and Mair, 1994; Zhang et al., 1998). The magnitude of the deficits due to MDT lesions is probably related to the extent of the lesions, to the task used, as well as to the difficulty of the task. In fact, Eichenbaum et al. (1980) reported that the major effects of MDT lesions appear when the task complexity is high. Interestingly, the difficulty level of the task may be linked to the attentional demand. An attention deficit may underlie the problems of rodents and humans with damage to the MDT to perform difficult odor discrimination tasks. The role of the MDT in olfactory attention was recently investigated in humans. Plailly et al. (2008) measured attention-dependent network activity using functional magnetic resonance imaging (fMRI). They observed that attending specifically to odor (compared to tone) increases the coupling between piriform cortex to MDT and MDT to orbitofrontal cortex. Corroborating these results, Veldhuizen and Small (2011) observed in humans, using fMRI, activation of the MDT in response to attention to odorants but not tastants. Those two studies support the involvement of the MDT in attention to odors and may be related to the fact that the MDT can be involved in prediction error signaling where the response magnitude of the MDT is significantly higher to unexpected compared to expected odor stimuli (Zelano et al., 2011; Olofsson et al., 2013).

These results obtained with functional imaging are further supported by lesions and neuropsychological studies (Tham et al., 2009, 2011a,b) For example, Tham et al. (2011b) tested whether the MDT was likely involved in top-down directed olfactory attention by using a Target Odor Search Test and showed that patients with damage to the MDT performed poorer verbal-based search than controls. All these studies in humans indicate a possible role of the MDT in olfactory attention processing. However, this idea was debated by Keller (2011) who proposed that the olfactory inputs to the MDT may not be sufficient to support shift of attention toward odors. The question about the contribution of the MDT in olfactory attention still remains open and other studies are required to disentangle it. For example, future studies can assess the impact of lesions of the MDT in rats performing an attention-related task, such as the one described in Ljubojevic et al. (2014) and measure not only their performance relative to controls but also their sampling duration and their latency to reply. Those studies can also assess how and when during the attention-related task the MDT is required by selectively and temporally inhibiting the MDT at different periods of the task using optogenetics.

Lastly, electrophysiological recordings of the MDT in behaving animals may also help to better characterize the temporal contribution of the MDT in olfactory perception and odor-guided behavior. Regarding electrophysiological recordings of the MDT in behaving animals, to the best of our knowledge, there is only one published study recording single-unit activity in the MDT in animals actively engaged in olfactory tasks [in rats: (Kawagoe et al., 2007); *nota bene*: in Yarita et al. (1980), monkeys were awake though odors were presented passively]. Kawagoe et al. (2007) recorded the MDT activity in an olfactory task requiring animals to discriminate odor cues associated with reward or not. They observed that 10% (13/121 units) of MDT neurons recorded in the central and medial subnuclei responded to the odor cue. Most of these cue-responsive neurons displayed odorant selectivity with a difference of activity between odor cues associated to the same reinforcement category. Combined with this sensory selectivity, the most remarkable effect was that cue-responsive MDT neurons showed strong response preference to cues associated with a reward, and those responses were plastic to extinction and relearning and always related to the reward contingency. The MDT is thus sensitive to stimulus-reward association. Interestingly, the basolateral amygdala and the olfactory tubercle, which project to the medial and central subnuclei, respectively, can also encode the associated outcome of odors, which could contribute to this MDT activity (Schoenbaum et al., 1998; Gadziola et al., 2015). Finally, we recently recorded MDT units in rats performing a two alternative odor discrimination task (Courtiol and Wilson, 2015) and observed that a subset of units were odor selective. Intermingled with this sensory function, we observed that the MDT units displayed activity prior to odor sampling, presumably anticipatory activity, and seemed to encode the choice direction-goal location associated with the odor. This study as well as the one by Kawagoe et al. (2007) emphasize that the MDT may encode both basic sensory as well as complex olfactory functions. This observation is not unique to the MDT as coding both basic sensory and complex olfactory functions has notably been reported in piriform cortex and orbitofrontal cortex (Schoenbaum and Eichenbaum, 1995; Feierstein et al., 2006). With these limited images of MDT function, many questions remain, most notably what are the specific contributions of the MDT in these functions compared to the piriform cortex or the orbitofrontal cortex and what is the MDT adding to olfactory processing?

## Conclusion and Perspectives

Despite the unusual anatomy of the olfactory pathway, an olfactory thalamus can be identified—the MDT receives direct input from various olfactory areas (Figure 1A). By virtue of this specific pathway, the thalamic contributions to olfaction are woefully unexplored. Lesion studies in the 1980’s followed by more recent humans studies have provided the first evidence of the involvement of the MDT in olfactory processing and suggest a role for the MDT in functions ranging from olfactory perception to attention. While this work provides a glimpse of the place of the MDT in olfaction, many questions, as described above, remain unanswered. For example: Is the MDT role in olfaction similar to the role of primary sensory relays such as the lateral geniculate nucleus? If so, can principles of thalamic function be generalized to all sensory systems? We hypothesize that not to be the case. In olfaction, the functions of “primary sensory thalamic relay” including sensory coding, gain control, and state-dependent modulation may be distributed between the olfactory bulb and the piriform cortex (Murakami et al., 2005; Kay and Sherman, 2007). Moreover, given: (1) the anatomical place of the MDT in the olfactory pathway (convergence of many olfactory inputs); (2) the implication of the MDT in higher order functions including olfactory attention; and (3) the MDT being a higher order thalamic relay, the MDT may then be viewed as a higher order olfactory thalamus rather than a primary sensory thalamic relay. Identifying the precise role of MDT in olfactory perception and odor-guided behavior may be an excellent avenue for exploring broader questions of higher-order thalamic function.

## Conflict of Interest Statement

The authors declare that the research was conducted in the absence of any commercial or financial relationships that could be construed as a potential conflict of interest.
